# Charge disproportionation and nano phase separation in $\textit{R}\mathrm{SrNiO}_{4}$

**DOI:** 10.1038/s41598-020-74884-2

**Published:** 2020-10-22

**Authors:** H. Guo, Z. W. Li, C. F. Chang, Z. Hu, C.-Y. Kuo, T. G. Perring, W. Schmidt, A. Piovano, K. Schmalzl, H. C. Walker, H. J. Lin, C. T. Chen, S. Blanco-Canosa, J. Schlappa, C. Schüßler-Langeheine, P. Hansmann, D. I. Khomskii, L. H. Tjeng, A. C. Komarek

**Affiliations:** 1 Max-Planck-Institute for Chemical Physics of Solids, Nöthnitzer Str. 40, 01187 Dresden, Germany; 2 Institute of Applied Magnetics, Key Lab for Magnetism and Magnetic Materials of the Ministry of Education, Lanzhou University, Lanzhou, 730000 People’s Republic of China; 3 National Synchrotron Radiation Research Center (NSRRC), 101 Hsin-Ann Road, Hsinchu, 30076 Taiwan; 4 ISIS Facility, STFC Rutherford Appleton Laboratory, Harwell Oxford, Didcot, OX11 0QX UK; 5 Forschungszentrum Jülich GmbH, Jülich Centre for Neutron Science at ILL, 71 avenue des Martyrs, 38000 Grenoble, France; 6 Institut Laue-Langevin, 71 avenue des Martyrs, 38000 Grenoble, France; 7 IKERBASQUE, Basque Foundation for Science, 48013 Bilbao, Basque Country Spain; 8 Donostia International Physics Center, DIPC, 20018 Donostia-San Sebastian, Basque Country, Spain; 9 European X-ray Free Electron Laser Facility GmbH, Holzkoppel 4, 22869 Schenefeld, Germany; 10 Helmholtz-Zentrum Berlin für Materialien und Energie GmbH, Albert-Einstein-Str. 15, 12489 Berlin, Germany; 11 Physics Institute II, University of Cologne, Zülpicher Str. 77, 50937 Cologne, Germany

**Keywords:** Electronic properties and materials, Magnetic properties and materials, Phase transitions and critical phenomena, Materials science, Physics

## Abstract

We have successfully grown centimeter-sized layered $\textit{R}\mathrm{SrNiO}_{4}$ single crystals under high oxygen pressures of 120–150 bar by the floating zone technique. This enabled us to perform neutron scattering experiments where we observe close to quarter-integer magnetic peaks below $\sim 77~\mathrm{K}$ that are accompanied by steep upwards dispersing spin excitations. Within the high-frequency Ni–O bond stretching phonon dispersion, a softening at the propagation vector for a checkerboard modulation can be observed. We were able to simulate the magnetic excitation spectra using a model that includes two essential ingredients, namely checkerboard charge disproportionation and nano phase separation. The results thus suggest that charge disproportionation is preferred instead of a Jahn–Teller distortion even for this layered $\mathrm{Ni}^{3+}$ system.

## Introduction

One of the very interesting classes of transition metal compounds showing quite unusual and rich properties are the rare earth (RE) perovskite nickelates $\textit{R}\mathrm{NiO}_{3}$, containing Ni ions in a nominal low-spin 3+ state in octahedral coordination. As was shown starting with the work of Torrance *et al.*^1^, for small RE ions there exist two transitions in these systems, from the high-temperature metallic orthorhombic state *Pbnm* to a low-temperature insulating monoclinic phase ${\text{P2}}_{1}/n$, accompanied by magnetic ordering at lower temperatures with a rather unusual magnetic structure with the wave vector Q = (1/4, 1/4, 1/4) (in pseudocubic notation), i.e. with ordering of the type $\uparrow \uparrow \downarrow \downarrow$ in all three directions. For $\mathrm{NdNiO}_{3}$ and $\mathrm{PrNiO}_{3}$ the magnetic transition and the structural transition merge into a common first order phase transition which simultaneously is a metal-insulator transition. And for the largest RE ion La, the rhombohedral $\mathrm{LaNiO}_{3}$ remains metallic down to the lowest temperatures, and was supposed to be paramagnetic until signatures of antiferromagnetic correlations, with about the same magnetic propagation vector Q = (1/4, 1/4, 1/4), have recently also been discovered in $\mathrm{LaNiO}_{3}$ single crystals grown under high oxygen pressure^2^.

It is remarkable that the Jahn–Teller distortion expected for the nominal low-spin configuration $t_{2g}^6e_g^1$ does not materialize in this $\textit{R}\mathrm{NiO}_{3}$ system. This was explained early on^3,4^ by the idea of charge disproportionation of the type $2\cdot \mathrm{Ni}^{3+}$ $\rightarrow$ $\mathrm{Ni}^{2+} + \mathrm{Ni}^{4+}$ which actually occurs rather on the ligands. The $\mathrm{Ni}^{3+}$ and even more so the $\mathrm{Ni}^{4+}$ in oxides have very small or negative charge transfer energies $\Delta (= \mathrm{E(d}^{(n+1)}2\mathrm{p}^5$) - $\mathrm{E(d}^n2\mathrm{p}^6$) )^5–7^, so that the charge disproportionation should be viewed rather as $2\cdot (\mathrm{Ni}^{2+}{\underline{L}}$) $\rightarrow$ $\mathrm{Ni}^{2+} + \mathrm{Ni}^{2+}{\underline{L}}^2$, where ${\underline{L}}$ stands for a ligand hole, which could then be termed as a valence bond disproportionation^8^. This picture explains the structural transitions in $\textit{R}\mathrm{NiO}_{3}$, at which inequivalent Ni ions appear^9^. Moreover, within the charge disproportionation scenario also the low-temperature magnetic structure of the $\uparrow \uparrow \downarrow \downarrow$ type^3^ is naturally explained. However, so far, only ‘ad-hoc’ configuration interaction models exist, and the phenomenon remains elusive from the point of view of *ab-initio* theories^10,11^. Note that the tendency to a charge, or valence bond disproportionation is a local property. However, there exists also an alternative picture^12,13^, relying mainly on the Fermi-surface properties of metallic nickelates, which is actually a collective, not local physics.

The interesting question now arises whether the charge or valence bond disproportionation still wins over the Jahn–Teller distortion for $\mathrm{Ni}^{3+}$ oxides where the local coordination is no longer $O_h$^14,15^. For example, the layered system $\textit{R}\mathrm{SrNiO}_4$ has a $\mathrm{K}_2\mathrm{NiF}_4$ crystal structure with $\mathrm{NiO}_6$ octahedra that are elongated along the tetragonal axis. In such a case would the charge disproportionation be absent and instead the Jahn–Teller $t_{2g}^6e_g^1$ be stabilized? Electronic structure calculations in the LDA+NMTO downfolding scheme predict the $3\mathrm{z}^2\mathrm{-}\mathrm{r}^2$ orbital to be more stable than the $\mathrm{x}^2\mathrm{-}\mathrm{y}^2$ orbital by about $\sim 0.26~\mathrm{eV}$^16–18^, which is a large energy difference regarding an optical gap of $\lesssim 0.2\mathrm{~eV}$. However, until now one could not prepare large high quality single crystals of these layered $\mathrm{Ni}^{3+}$ 2-1-4-nickelates. $\textit{R}_{2-x}\mathrm{Sr}_{x}\mathrm{NiO}_{4}$ have so far been studied only for doping levels *x* away from the $x=1$ pure $\mathrm{Ni}^{3+}$ situation. It is known, for example, that for $x \ll 1$ there is $\mathrm{Ni}^{2+}/\mathrm{Ni}^{3+}$ charge order which modulates the spin structure^19–21^. At half-doping, i.e. for *x* = 0.5, a $\mathrm{Ni}^{2+}$/$\mathrm{Ni}^{3+}$ checkerboard charge ordering occurs^22^ similar to the isostructural cobaltates^23–28^. This checkerboard charge order survives up to ~70% of hole-doping^29^ and the materials stay insulating.

Some initial studies on single crystals of highly hole-doped $\textit{R}_{2-x}\mathrm{Sr}_x\mathrm{NiO}_4$ exist and show an about equal population of four different kind of states around $x~\sim ~1$ : $\mathrm{Ni}^{2+}$, $\mathrm{Ni}^{3+}$ with $3\mathrm{z}^2$-$\mathrm{r}^2$ orbital occupation, $\mathrm{Ni}^{3+}$ with $\mathrm{x}^2$–$\mathrm{y}^2$ orbital occupation and $\mathrm{Ni}^{4+}$^30^. Moreover, also ARPES studies on these materials exist^16,17^.

However, so far, no detailed study of the physical properties of the compounds with the pure $\mathrm{Ni}^{3+}$ oxidation state have been carried out and it is unknown whether the Jahn–Teller orbital occupation can be stabilized by the strongly distorted Ni–oxygen environment instead of the $\mathrm{Ni}^{2+}/\mathrm{Ni}^{4+}$ or $\mathrm{Ni}^{2+}/\mathrm{Ni}^{2+}{\underline{L}}^2$ charge disproportionation. We note that the mechanism for such a charge disproportionation is quite distinct from that for a $\mathrm{Ni}^{2+}/\mathrm{Ni}^{3+}$ charge ordering since a disproportionation in a stoichiometric system involves an energy term related to the on-site Coulomb interaction *U* (which needs to be compensated by another interaction energy), while for ordering of charge in a non-stoichiometric or doped material the term *U* is not operative.

**Figure 1:**
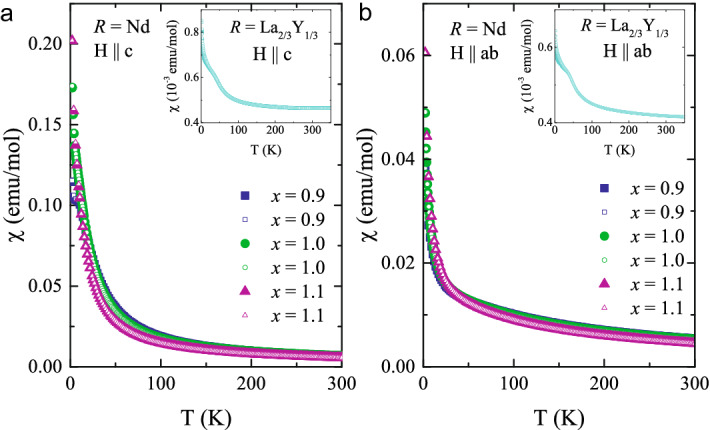
**Magnetic susceptibility**—Temperature dependence of the magnetic susceptibility $\chi$ for $\mathrm{Nd}_{2-x}\mathrm{Sr}_{x}\mathrm{NiO}_{4}$ (in the main panel) and $\mathrm{La}_{2/3}\mathrm{Y}_{1/3}\mathrm{SrNiO}_{4}$ (in the inset). Solid/open symbols denote ZFC /FC measurements. Note, that the magnetic susceptibility of $\mathrm{NdSrNiO}_{4}$ is governed by the $\mathrm{Nd}^{3+}$ ions, which masks the signal from the Ni moments.

**Figure 2:**
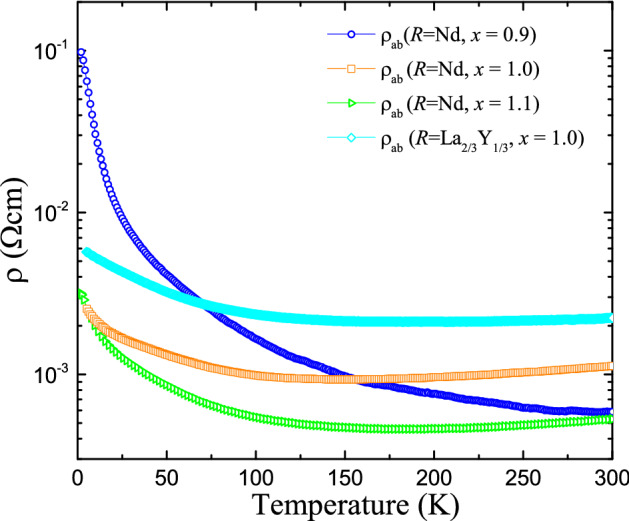
**Electrical resistivity**—Temperature dependence of the in-plane resistivity $\rho _{ab}(T)$ for $\mathrm{Nd}_{2-x}\mathrm{Sr}_{x}\mathrm{NiO}_{4}$ and $\mathrm{La}_{2/3}\mathrm{Y}_{1/3}\mathrm{SrNiO}_{4}$.

**Figure 3:**
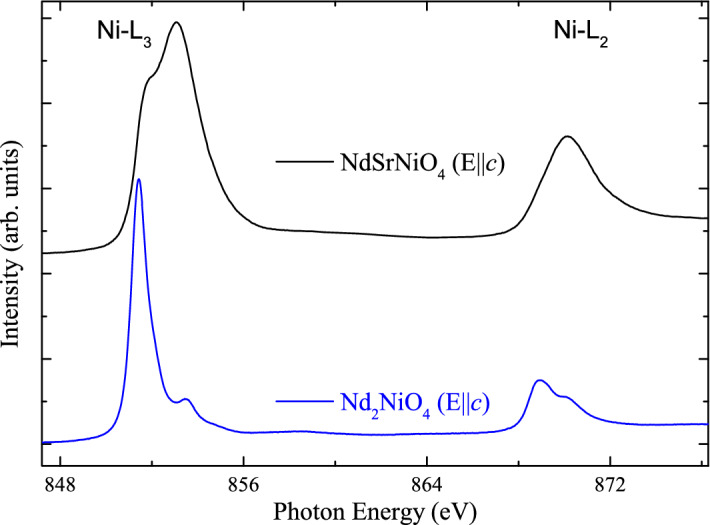
**X-ray absorption spectroscopy **—$\mathrm{Ni-L}_{2,3}$ XAS spectra of the $\mathrm{NdSrNiO}_{4}$ single crystal and—for comparison—a $\mathrm{Nd}_{2}\mathrm{NiO}_{4}$ single crystal measured at 300 K.

## Results

Powder X-ray diffraction shows that our $\mathrm{NdSrNiO}_{4}$ samples are impurity-free. From the refinement of the powder X-ray diffraction data we obtain a *c*/*a* ratio that amounts to $\sim$3.26 and apical and basal Ni–O distances which amount to 2.058(8) Å and 1.895(1) Å respectively, thus, indicating a strongly tetragonaly distorted oxygen environment with an apical-to-basal distance ratio of 1.09.

The magnetic susceptibility in $\mathrm{NdSrNiO}_4$ is dominated by the $\mathrm{Nd}^{3+}$ moments, see Fig. 1. For $\mathrm{La}_{2/3}\mathrm{Y}_{1/3}\mathrm{SrNiO}_{4}$ with non-magnetic *R*-ions the effective moment of Ni can be determined and amounts to $\sim 0.23~\mu_{\mathrm{B}} /{\mathrm{Ni}}$ according to Curie–Weiss fits.

The temperature dependence of the in-plane resistivity is shown in Fig. 2. A bad metallic behavior can be observed at high temperatures followed by a semiconducting temperature dependence at lower temperatures, thus, indicating a (gradual) metal–semiconductor transition around $\sim 150~\mathrm{K}$.

The $\mathrm{Ni-L}_{2,3}$ X-ray absorption spectrum of our $\mathrm{NdSrNiO}_{4}$ single crystal is shown in Fig. 3 and is compared to that of our $\mathrm{Nd}_2\mathrm{NiO}_{4}$ single crystal serving as a $\mathrm{Ni}^{2+}$ reference compound. It is well known that the XAS spectra at the $\mathrm{L}_{2,3}$ edge of transition metals are highly sensitive to the valence state^31–33^—an increase of the valence state of the transition metal ion by one causes a shift of the XAS $\mathrm{L}_{2,3}$ spectra by one or more eV towards higher energies. The more than one eV higher energy shift from $\mathrm{Nd}_2\mathrm{NiO}_{4}$ to $\mathrm{NdSrNiO}_{4}$ indicates the formal $\mathrm{Ni}^{2+}$ and $\mathrm{Ni}^{3+}$ valence states for the former and the latter compound respectively. Here we would like to note that the $\mathrm{NdSrNiO}_{4}$ spectrum cannot be interpreted in terms of an ionic Ni $3\mathrm{d}^{7}$ configuration, but, rather by a coherent mixture of $3\mathrm{d}^{8}$ and $3\mathrm{d}^{8}{\underline{L}}^2$ configurations^3,4,7,34^, where each ${\underline{L}}$ denotes a hole in the oxygen ligand. We can exclude any Ni$^{2+}$ impurities in our $\mathrm{NdSrNiO}_{4}$ single crystal, otherwise the sharp main peak of Ni$^{2+}$ impurity spectrum would have been visible as a sharp shoulder at the leading edge.

**Figure 4:**
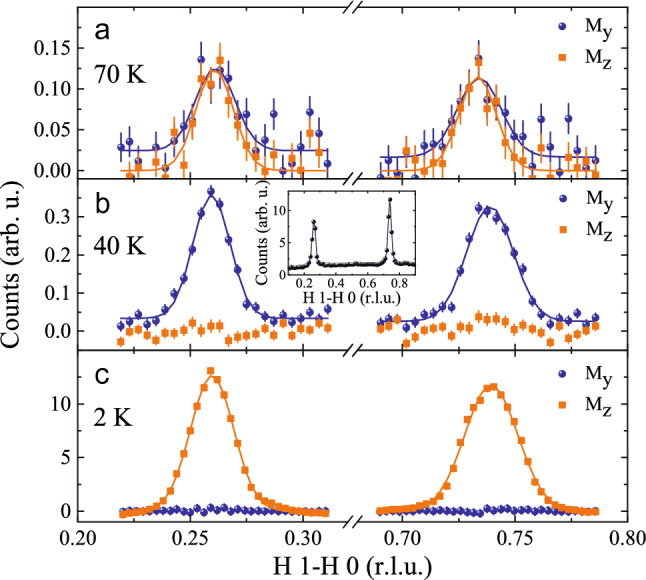
**Neutron diffraction**—Elastic, polarized neutron scattering measurements of $\mathrm{NdSrNiO}_{4}$ measured at the IN12 spectrometer showing the extracted $M_y$ and $M_z$ components for [*H*, 1-*H*, 0]—scans at different temperatures of (**a**) 70 K, (**b**) 40 K and (**c**) 2 K. The solid curves are Gaussian fits. The inset in (**b**) shows unpolarized neutron scattering intensities measured at the IN8 spectrometer within a larger range.

**Figure 5:**
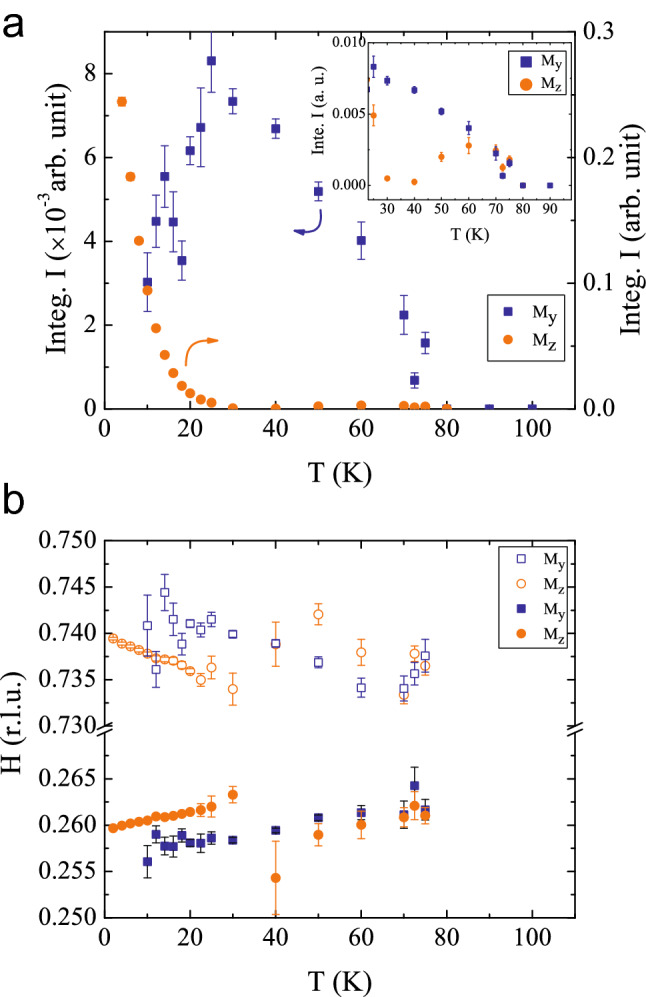
**Analysis of polarized neutron data**—Fitting results for $\mathrm{NdSrNiO}_{4}$. (**a**) Temperature dependence of the integrated intensities for the $M_y$ and $M_z$ components for the peak near (0.25, 0.75, 0). Note the different scales for the two components. The inset shows an enlargement of the high temperature regime. (**b**) Temperature dependence of the peak position at (*H* 1-*H* 0).

**Figure 6:**
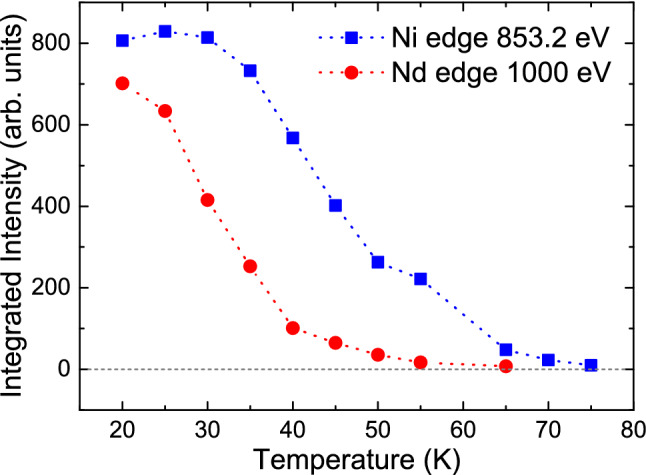
**Resonant X-ray diffraction**—Temperature dependence of the magnetic intensities at the $\mathrm{Nd-}M_5$ and Ni-$L_3$ resonances.

**Figure 7:**
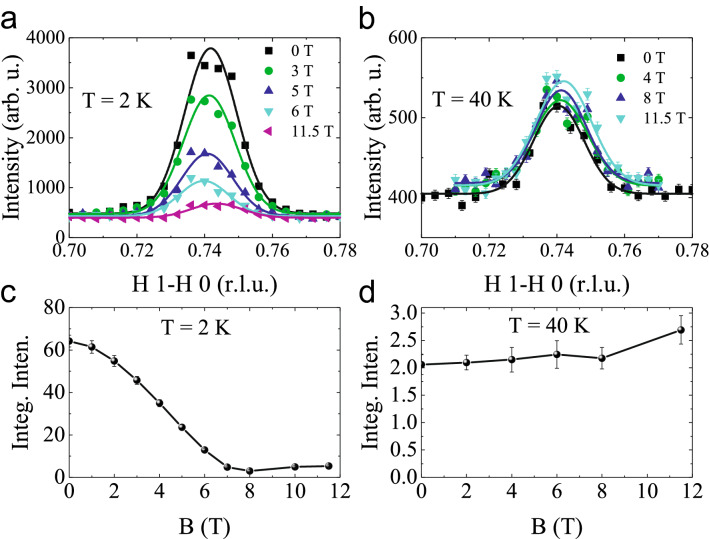
**Magnetic field dependence**—Magnetic field dependence of the neutron intensites measured along the [*H*, 1-*H*, 0] direction at (**a**) 2 K and (**b**) 40 K in $\mathrm{NdSrNiO}_{4}$. (**c**,**d**) The magnetic field dependence of the integrated intensities at 2 and 40 K, respectively.

Due to the availability of large single crystals we are now able to perform (polarized) neutron scattering experiments on $\textit{R}\mathrm{SrNiO}_{4}$. As shown in Fig. 4, at low temperatures (i.e. below $\mathrm{T}_N~\sim 80~\mathrm{K}$) magnetic peaks could be detected around quarter-integer positions within the *H* *K* 0 plane of reciprocal space. The magnetic origin of these peaks can be confirmed by polarization analysis and the extracted $M_y$ and $M_z$ components are shown in Fig. 4. Gaussian functions are fitted to the data. In $\mathrm{NdSrNiO}_{4}$ the Ni moments start ordering below $\sim 77~\mathrm{K}$, see Fig. 5. Close to 70 K, the intensities of the $M_y$ and $M_z$ components are comparable, thus, indicating that the magnetic moments are canted out of the *ab* plane first. But, on further cooling below 60 K the $M_y$ component increases while the $M_z$ component almost vanishes, thus, demonstrating that the magnetic moments become confined to the *ab* plane. For $\mathrm{NdSrNiO}_{4}$ the $M_z$ component increases substantially on further cooling below 30 K. This can be attributed to the ordering of the Nd moments in *c* direction at lowest temperatures. The resulting values for fitted intensities and peak positions are plotted in Fig. 5.

Additionally, we measured also the temperature dependence of the Nd- and Ni-contribution of the antiferromagnetic reflections by means of resonant soft X-tray diffraction^35^, see Fig. 6. These element selective measurements corroborate our findings that the Nd-moments start to order at distinctly lower temperatures than the Ni-moments. Note, that resonant X-rays are element specific and not probing the same magnetic intensity as neutrons are doing. Also short range correlations will be less integrated in this measurement due to the higher resolution which might result in a different temperature dependence compared to the neutron results.

As shown in Fig. 7a and b, the magnetic fields suppress the magnetic reflection intensities drastically at 2 K, while having almost no influence at 40 K, which can be seen more clearly from the extracted magnetic field dependence of the integrated intensities using a Gaussian fit to the data, as shown in Fig. 7c and d. These results reflect the full polarization of the $\mathrm{Nd}^{3+}$moments below the Nd-magnetic ordering temperatures in fields above 6 T whereas the (much smaller) Ni magnetic moments (that are observable at 40 K) are much less affected.

Summarizing, distinctly below $\mathrm{T}_N$ (e.g. around 40 K) the Ni magnetic moments are aligned within the *ab* planes. For $\mathrm{NdSrNiO}_{4}$ also the Nd moments start ordering below roughly 20 K with the larger Nd moments being aligned in the *c*-direction. The observation of quarter integer magnetic peaks in $\textit{R}\mathrm{SrNiO}_{4}$ is compatible with a charge disproportionation of the nominal $\mathrm{Ni}^{3+}$ ($3\mathrm{d}^7$) ions into a $3\mathrm{d}^{8}/3\mathrm{d}^{8}{\underline{L}}^2$ configurations. The ordering of these charges is in a checkerboard pattern.

**Figure 8:**
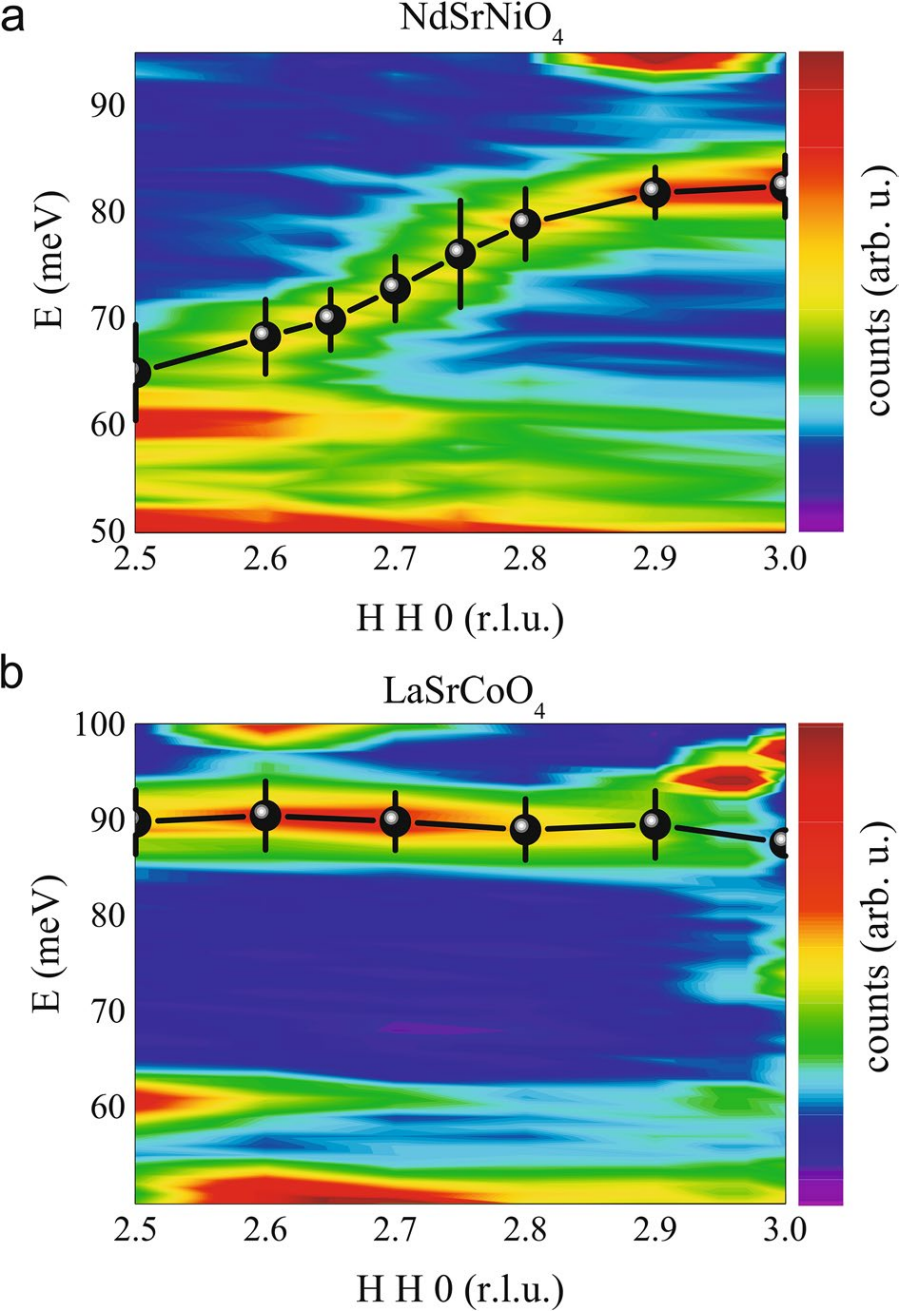
**Bond stretching Phonons**—Phonon dispersions at 2 K measured at the IN8 spectrometer for (**a**) $\mathrm{NdSrNiO}_{4}$ and (**b**) $\mathrm{LaSrCoO}_4$.

This kind of charge ordering is also in agreement with our inelastic neutron measurements of the longitudinal Ni–O bond stretching phonon mode, compare also Ref.^23^. For $\mathrm{high-T}_C$ superconducting cuprates it is known that such phonon softening of the Cu–O bond stretching phonon modes has been observed at the propagation vector of the underlying charge (stripe) order^36^, i.e. one would expect a bond-stretching phonon anomaly at the propagation vector of the underlying charge ordering propagation vector. As can be seen in Fig. 8a, this high-frequency phonon dispersion softens for $\mathrm{NdSrNi}^{3+}\mathrm{O}_{4}$ at half-integer propagation vectors similar to that in *half-doped* cobaltates with very robust checkerboard charge order^23^. In contrast to that, the same measurement of the *pure* $\mathrm{Co}^{3+}$ reference material $\mathrm{LaSrCo}^{3+}\mathrm{O}_4$—see Fig. 8b—reveals no such phonon softening at the zone boundary i.e. towards $\mathbf{Q} = (2.5~2.5~0)$.

In $\mathrm{NdSrNiO}_{4}$ the static distortions implied by this kind of charge distribution could not be detected in analogous elastic neutron measurements. However, these distortions might be too weak to be detectable possibly because of small distortions that are associated with the charge disproportionation, since the charges are delocalized towards the oxygen ions. A similar situation might occur in $\mathrm{LaNiO}_3$^2^. Note that the soft X-rays are unable to reach the required points in reciprocal space and that resonant hard X-ray scattering is unfavorable since any gain in intensity at the K-edge is marginal and will be overcompensated by the effects of fluorescence.

**Figure 9:**
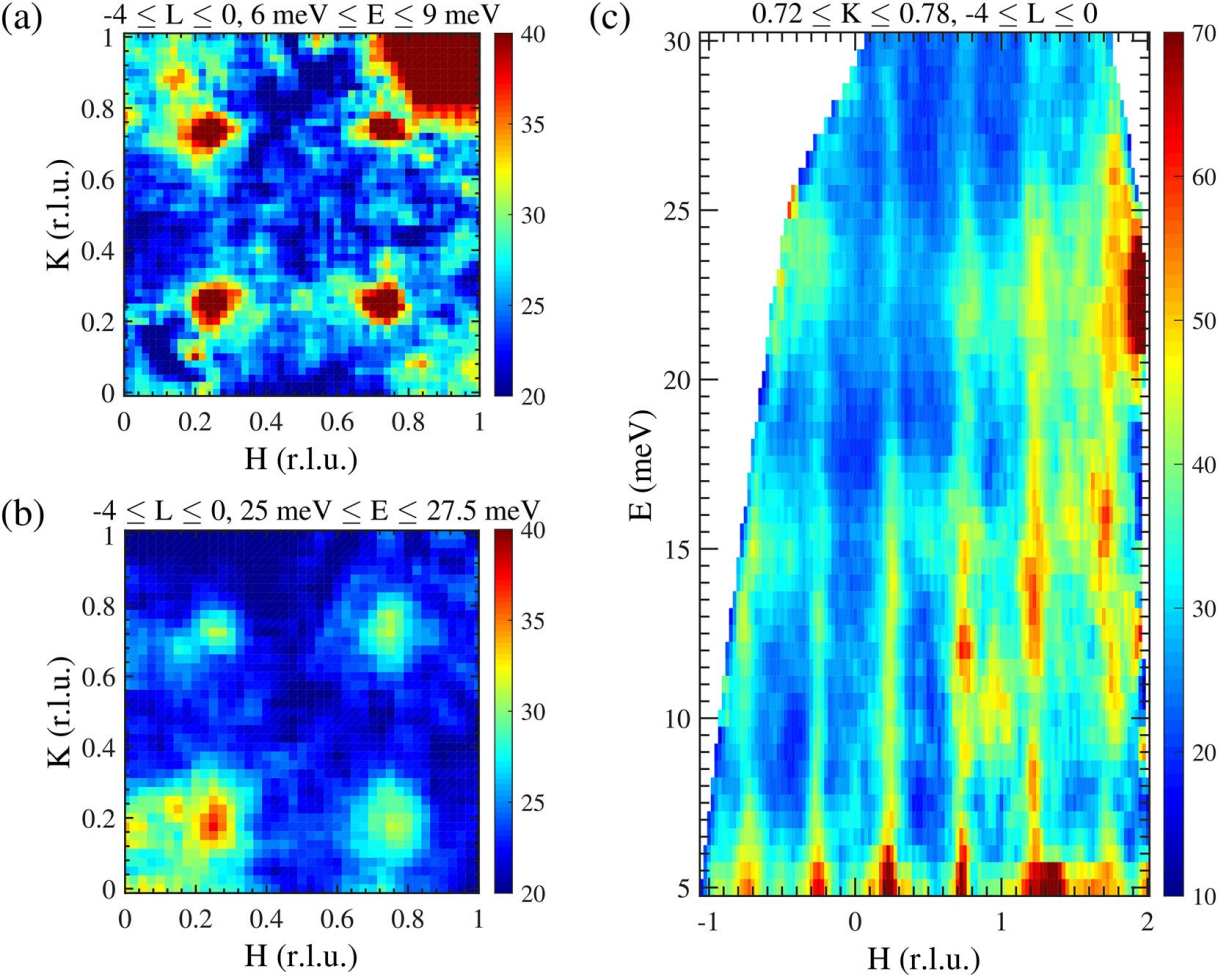
**Spin excitations**—Magnetic excitation spectra of $\mathrm{La}_{2/3}\mathrm{Y}_{1/3}\mathrm{SrNiO}_4$ measured at the TOF spectrometer MERLIN at 6 K.

**Figure 10:**
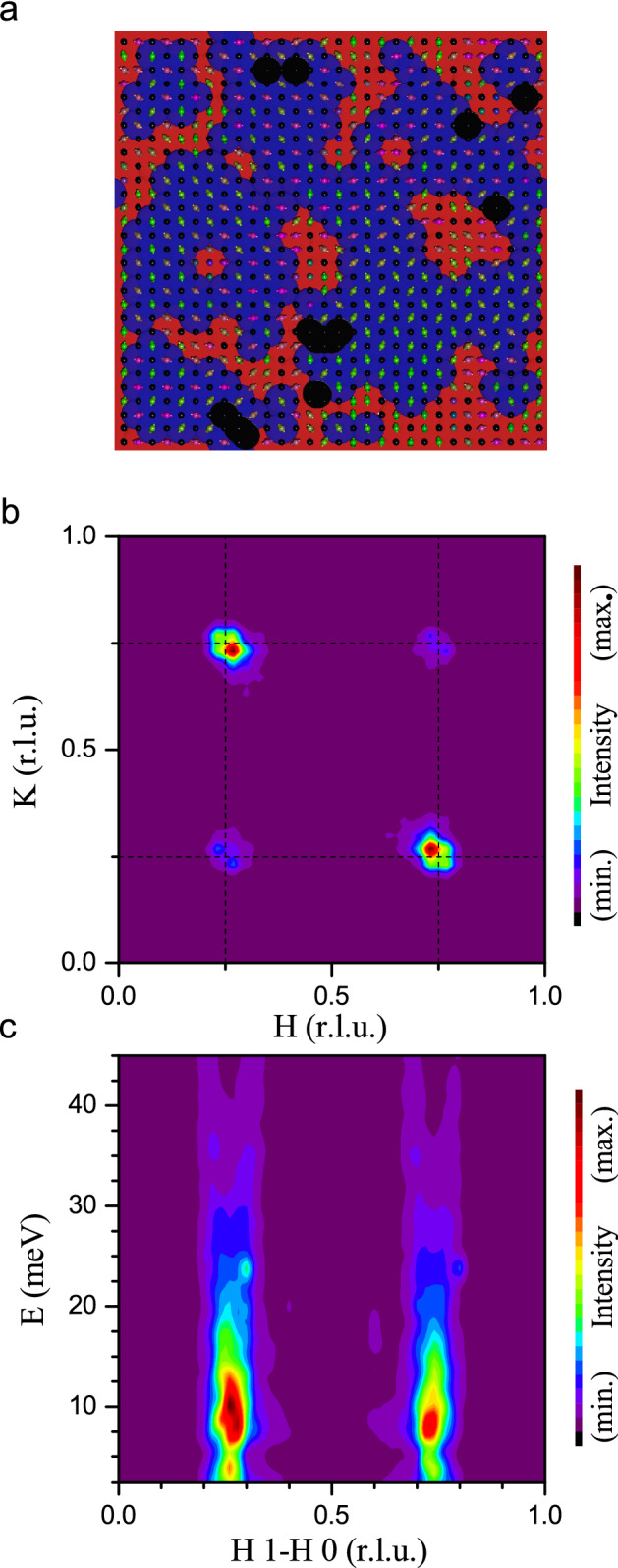
**Nano phase separation model**—Spin wave simulations of $\textit{R}\mathrm{SrNiO}_{4}$ within a nano phase separation scenario. (**a**) One of the four $30\times 30$ meshes obtained from MonteCarlo simulations with a similar algorithm as used for checkerboard charge ordered cobaltates in Ref.^24^. In (**b**) and (**c**) the obtained elastic and inelastic neutron scattering intensities which have been calculated and averaged for four meshes are shown. Black/colored spheres: $\mathrm{Ni}^{4+}-\mathrm{ions/Ni}^{2+}\mathrm{-ions}$. In this model only the $\mathrm{Ni}^{2+}$-ions are considered since these are the only ions with sizable nn, nnn, etc. exchange interactions among each other. The red/blue/black areas indicate regions which are hosting large/small/no exchange interactions $J/J'/0$. The incommensurability arises from frustration due to nano phase separation.

Finally, we measured the magnetic excitation spectrum of $\mathrm{La}_{2/3}\mathrm{Y}_{1/3}\mathrm{SrNiO}_4$ at 6 K (at the MERLIN time-of-flight spectrometer with an incident energy of 41 meV), see Fig. 9. These measurements show that these nickelates are different from the usual magnets—an upward dispersion becomes apparent which strongly resembles the one in highly hole-doped cobaltates^26^, see Fig. 9c. In the cobaltates this excitation spectrum could be explained by a nano phase separation model^24^ with two relevant exchange interactions—one between two $\mathrm{Co}^{2+}$-ions accross a hole ($J'$) and a much weaker one across two or more $\mathrm{Co}^{3+}$-ions ($J''$ etc.). Due to the similarity with the cobaltates, one might think of a similar model for the nickelates, but now with larger values of the exchange interactions that will scale the entire spectrum to higher energies. So we assume a fully charge disproportionated nickelate system with disorder, where we take the nearest neighbor exchange interactions between the magnetic Ni ions to be *J* $=$ $J^{[100]}$ $=$ − 30 meV, and the exchange interaction across a non-magnetic Ni ion to be $J'=J^{[200]}=-10$ meV, plus also a small (and less important) diagonal exchange interaction $J^{[110]}=-2$ meV. Using the *McPhase* program code we calculated the magnetic ground state and the corresponding spin wave excitations for four $30\times30$ meshes—one of them shown in Fig. 10a displaying domains of several nanometer in size. The corresponding elastic and inelastic neutron scattering intensities of the spin correlations are shown in Fig. 10b,c. The incommensurate magnetic peaks appear at almost the same positions as observed in the experiment. Furthermore, also the magnetic excitation spectrum strongly resembles the experimental data, compare Figs. 9 and 10c. Hence, a nano phase separation model for a charge disproportionated sample is able to simulate the elastic and inelastic neutron scattering measurements in $\textit{R}\mathrm{SrNiO}_{4}$. In our model, nano phase separation is not driven by carrier doping but by disorder within a completely charge disproportionated sample which inevitably leads to the creation of nanoscopic regions with large exchange interactions *J* (red areas in Fig. 10a) interspersed with regions with small exchange interactions $J'$ (blue areas in Fig. 10a) and regions with small exchange interactions that can be neglected (black areas in Fig. 10a). Such a scenario might be also applicable to other systems with checkerboard charge order with a certain degree of disorder. Indications for nanoscale phase separation have recently also been reported for the high-temperature superconducting cuprates^37,38^ which points to a possibly significant role of these effects for the physical properties of these systems.

## Conclusion

We succeeded in synthesizing stoichiometric $\mathrm{Ni}^{3+}$ 2-1-4 nickelate single crystals by the high-pressure floating zone technique. We found that this system prefers to undergo a charge disproportionation, although it crystallizes in a tetragonal structure with strongly distorted oxygen environment of the Ni ions that would allow for a Jahn–Teller effect with an occupation of the $3z^2-r^2$ orbital. This is the more remarkable when one considers the fact that the effective crystal field from theory places the $3z^2-r^2$ orbital $\sim 0.26~\mathrm{eV}$ below the $x^2-y^2$ which is a large energy difference. Hence, the experimental results suggest that the negative charge transfer character of the $\mathrm{Ni}^{3+}$ ion in these oxides provides a very strong drive for charge disproportionation to occur of the type $2\cdot \mathrm{Ni}^{2+}{\underline{L}}\rightarrow \mathrm{ Ni}^{2+} +\mathrm{Ni}^{2+}{\underline{L}}^2$, leading to the formation of the observed checkerboard magnetic superstructure and overcoming the tendency to Jahn–Teller distortion. Alternatively, one might also think in terms of Fermi surface effects in view of the close proximity to a metallic state in this 2-1-4 material. In any case, it is now fully understandable that the 1-1-3 nickelates undergo charge disproportionation since the cubic crystal structure does not provide *a priori* a $3\mathrm{z}^2\mathrm{-r}^2 / \mathrm{x}^{2}\mathrm{-y}^2$ crystal field splitting that would have helped the Jahn–Teller stabilization. $\textit{R}\mathrm{SrNiO}_{4}$ can thus serve as a bench mark system for further theory development to describe properly the role of holes in the oxygen band. Finally, the magnetic correlations and the magnetic excitation spectra in these nickelates can be explained by a nano phase separation model which is different from usual magnets with long range magnetic order and points to the possible significance of such nano phase separation for understanding the physical properties of these materials.

## Methods

Single crystals of $\mathrm{NdSrNiO}_{4}$, $\mathrm{La}_{2/3}\mathrm{Y}_{1/3}\mathrm{SrNiO}_{4}$ and $\mathrm{Nd}_2\mathrm{NiO}_{4}$ were grown by the floating zone technique using a high pressure mirror furnace from Scidre. Here, we studied two $\mathrm{Ni}^{3+}$ systems: $\mathrm{NdSrNiO}_{4}$ and $\mathrm{La}_{2/3}\mathrm{Y}_{1/3}\mathrm{SrNiO}_{4}$. The former one because for *R*=Nd there is no undesirable mixture of $\mathrm{Ni-L}_{3}$ and $\mathrm{La-M}_{4}$ edges in the XAS spectra, the latter one because Nd has an undesirable large magnetic moment that might easily overshadow the Ni contribution. We note that the pure La system was more difficult to grow as large single crystals than the Y-substituted compound. The $\mathrm{Nd}_2\mathrm{NiO}_{4}$ was grown to serve as a $\mathrm{Ni}^{2+}$ reference system with the same crystal structure as the $\mathrm{NdSrNiO}_{4}$ and $\mathrm{La}_{2/3}\mathrm{Y}_{1/3}\mathrm{SrNiO}_{4}$. In order to perform floating zone growth for $\mathrm{Nd}_{2-x}\mathrm{Sr}_x\mathrm{NiO}_{4}$ ($x~=0.9$, 1.0 and 1.1) and $\mathrm{La}_{2/3}\mathrm{Y}_{1/3}\mathrm{SrNiO}_{4}$ starting materials of $R_2\mathrm{O}_{3}$, $\mathrm{SrCO}_{3}$ and NiO were mixed in appropriate ratios and ground thoroughly in an agate mortar followed by sintering steps at $1000^{\circ }\mathrm{C-}1200^{\circ }\mathrm{C}$ in air for several days with intermediate grindings. The obtained composition was then pressed under $\sim 100~\mathrm{MPa}$ hydrostatic pressure into a rod of about 6–8 mm in diameter and 120(20) mm in length, which was subsequently sintered at $1000^{\circ }\mathrm{C}$ in air. The floating zone growth was performed with a growth rate of 2 mm/h in a flowing $\mathrm{O}_2$ atmosphere ($\sim 0.2\,\mathrm{l/min}$) at a pressure of 100 bar, 120  bar and 150 bar for $x=0.9$, 1.0 and 1.1, respectively.

Phase purity was confirmed by powder X-ray diffraction (XRD) measurements on ground single crystals with a $2\theta$ step of 0.005$^o$ using Cu $K_{\alpha 1}$ radiation of a laboratory X-ray source. It was possible to describe the crystal structure properly with space group *I4/mmm* for all grown samples. The refined lattice parameters and the resulting unit cell volumes are listed in Table 1. The linear change of the lattice parameters as a function of *x* further confirms the successful substitution of the Nd atom by Sr atom within the $\mathrm{Nd}_{2-x}\mathrm{Sr}_x\mathrm{NiO}_{4}$ series. This is also corroborated by inductively coupled plasma optical emission spectrometry (ICP-OES) measurements, see the value of *x*(ICP) in Table 1. Moreover, thermogravimetric measurements confirmed that the oxygen content is close to the nominal value, see Table 1.

**Table 1:** Growth condition and physical parameters of $\mathrm{Nd}_{2-x}\mathrm{Sr}_x\mathrm{NiO}_{4}$ and $\mathrm{La}_{2/3}\mathrm{Y}_{1/3}\mathrm{SrNiO}_{4}$: $v_{G}$ is the growth speed, $\mathrm{O}_2$ pressure for the crystal growth, lattice parameters *a*, *b*, *c* and unit cell volume from the Rietveld refinements, $\mu _{eff}^{c}/\mu _{eff}^{ab}$ and $\theta _{CW}^{c}/\theta _{CW}^{ab}$ are effective moment per formula unit and Curie–Weiss temperature determined from the Curie–Weiss fit $\chi (T) = \chi _0 + C/(T-\theta _{CW})$ to the magnetic susceptibility.

Sample	*x* = 0.9	*x* = 1.0	*x* = 1.1	$La_{2/3}$Y$_{1/3}$
$v_{G}$ (mm/h)	2	2	2	2
O$_2$ pressure (bar)	100	120	150	150
*a*, *b* (Å)	3.7850(3)	3.7890(3)	3.7956(3)	3.7990(3)
*c* (Å)	12.352(1)	12.322(1)	12.278(1)	12.356(1)
Volume (Å$^3$)	176.96(3)	176.90(2)	176.89(2)	178.318(3)
$\mu _{eff}^{c}$ ($\mu _B$)	3.928(6)	3.777(3)	3.532(6)	0.165(1)
$\mu _{eff}^{ab}$ ($\mu _B$)	4.364(4)	3.942(5)	3.701(9)	0.229(1)
$\theta _{CW}^{c}$ (K)	$-$6.2(3)	$-$6.2(1)	$-$10.3(3)	25(1)
$\theta _{CW}^{ab}$ (K)	$-$134.3(3)	$-$105.8(4)	$-$92.3(6)	$-$26(1)
$x(\mathrm {ICP})$	0.90(2)	1.01(2)	1.12(2)	
$\delta (\mathrm {TG})$	0.02(2)	0.00(2)	$-$0.01(2)	

$x(\mathrm {ICP})$ is measured by ICP-OES, $\delta (\mathrm {TG})$ is measured by thermogravimetric (TG) analysis.

Magnetic susceptibility ($\chi$) measurements have been performed using a Quantum Design vibrating sample magnetometer (VSM) and the electrical resistivities have been measured with a four-probe method using the Quantum Design PPMS system.

The $\mathrm{Ni-L}_{2,3}$ X-ray absorption spectroscopy (XAS) measurements have been performed at the 11A beamline of the National Synchrotron Radiation Research Center (NSRRC), Taiwan. A NiO single crystal was measured simultaneously for energy calibration. The photon energy resolution at the Ni $\mathrm{L}_{2,3}$ edges was set at 0.3 eV. The spectra were recorded at 300 K using the total electron yield method.

Unpolarized elastic neutron scattering measurements were performed on the IN8 spectrometer at the Institut Laue Langevin (ILL) using PG monochromator and PG analyzer with fixed $k_f=2.662$Å^−1^ and two PG filters for the suppression of higher order contaminations. Unpolarized inelastic neutron scattering measurement were performed on the IN8 spectrometer at the ILL and on the MERLIN time-of-flight (TOF) spectrometer at ISIS^39,40^. For the IN8 measurement, doubly focused Cu monochromator and PG analyzer were used with two PG filters. The MERLIN experiment has been performed in repetition-rate multiplication (RRM) mode. Longitudinal polarized elastic neutron scattering measurements were performed on the IN12 spectrometer at the Institut Laue-Langevin (ILL) equipped with double focusing pyrolythic graphite (PG) monochromator and Heusler analyzer. The beam was polarized by a transmission polarizer in the neutron guide. The monochromator was set for a wave vector of $2.25$Å^−1^, and, a velocity selector was used for suppression of higher order contamination. The flipping ratio amounts to $\sim 22.2$. For the polarization analysis, the *x* axis is defined along the direction of **Q**, the *y* axis is perpendicular to **Q** and within the scattering plane, and the *z* axis is perpendicular to the scattering plane. Note, that we used the tetragonal setting for all our neutron measurements with lattice constant $a = b \sim 3.79$Å and $c \sim 12.4$Å.

Resonant soft X-ray diffraction at the Nd $3d \rightarrow 4f$ ($M_5$, 1000 eV) and Ni $2p \rightarrow 3d$ ($L_3$, 853.2 eV) resonances have been measured at the UE56/2-PGM1 beam line at BESSY II. The data were recorded in horizontal scattering geometry with the X-rays linear polarized in the scattering plane ($\pi$-polarization). The scattered photons were detected with an in-vacuum CCD camera.

Magnetic field dependence of the antiferromagnetic correlations in $\mathrm{NdSrNiO}_{4}$ was measured on the D23 diffractometer with magnetic fields applied vertically, i.e., out of the *HK*0 scattering plane.
